# Molecular detection of *Babesia microti* in dromedary camels in Egypt

**DOI:** 10.1007/s11250-023-03507-5

**Published:** 2023-02-20

**Authors:** Radwa Ashour, Dalia Hamza, Mona Kadry, Maha A. Sabry

**Affiliations:** Department of Zoonoses, Faculty of Veterinary Medicine, Cairo University, P.O. Box 12211, Giza, Egypt

**Keywords:** *Babesia microti*, Beta-tubulin gene, Nested PCR, *18S* rRNA gene, *Babesia* spp., Camels

## Abstract

*Babesia* *microti* (Apicomplexa: Piroplasmida) causes a medically important tick-borne zoonotic protozoan disease. Egyptian camels are susceptible to *Babesia* infection; however, just a few cases have been documented. This study aimed to identify *Babesia* species, specifically *Babesia microti*, and their genetic diversity in dromedary camels in Egypt and associated hard ticks. Blood and hard tick samples were taken from 133 infested dromedary camels slaughtered in Cairo and Giza abattoirs. The study was conducted from February to November 2021. The *18S* rRNA gene was amplified by polymerase chain reaction (PCR) to identify *Babesia* species. Nested PCR targeting the β-tubulin gene was used to identify *B. microti*. The PCR results were confirmed by DNA sequencing. Phylogenetic analysis based on the ß-tubulin gene was used to detect and genotype *B. microti*. Three tick genera were identified in infested camels (*Hyalomma*, *Rhipicephalus*, and *Amblyomma*). *Babesia* species were detected in 3 out of 133 blood samples (2.3%), while *Babesia* spp. were not detected in hard ticks by using the *18S* rRNA gene. *B. microti* was identified in 9 out of 133 blood samples (6.8%) and isolated from *Rhipicephalus annulatus* and *Amblyomma cohaerens* by the β-tubulin gene. The phylogenetic analysis of the β-tubulin gene revealed that USA-type *B. microti* was prevalent in Egyptian camels*.* The results of this study suggested that the Egyptian camels may be infected with *Babesia* spp. and the zoonotic *B. microti* strains, which pose a potential risk to public health.

## Introduction

Hard ticks (Acari: Ixodidae) are hematophagous ectoparasites that can infest almost all vertebrates and transmit various diseases; as a result, ticks are regarded as one of the most important vectors of human and animal infections globally (Li et al. 2020). Tick-borne diseases are spreading globally due to tick population growth, geographic expansion, and human travel and commerce (Gratz 2006; Kim et al. 2021; Randolph and Rogers 2010).

Babesiosis is an emerging tick-borne zoonotic infectious disease of veterinary and medical importance and became a nationally notifiable disease in 2011 in the USA (CDC 2012; Motevalli Haghi et al. 2014). Babesiosis is caused by apicomplexan intraerythrocytic parasites of the genus *Babesia*; they are transmitted to vertebrates (ruminants, dogs, cats, birds, rodents, and humans) by ticks (Kalani et al. 2012; Mirahmadi et al. 2022). This disease can also be transmitted by blood transfusion, organ donation, and even congenitally (Herwaldt et al. 2011; Vannier and Krause 2012). The main vectors of *Babesia* spp. are several genera of the Ixodidae family as *Rhipicephalus*, *Ixodes*, *Haemaphysalis*, and *Hyalomma.* Geographically, *Ixodes scapularis* transmits *Babesia* parasites to natural hosts and also humans in the USA, *Ixodes ricinus* in Europe, and *Ixodes persulcatus* in Asia (Zamoto-Niikura et al. 2016).

Dromedaries are not resistant to *Babesia* infection. Babesiosis in camels causes anemia, fever, icterus, hemoglobinuria, and gastrointestinal stasis; pathogenicity varies according to *Babesia* species (Swelum et al. 2014). Factors that contribute to the increased risk of babesiosis include the importation of camels from areas with high infection rates and the spreading of vector ticks (Mirahmadi et al. 2022). Recent research by Salman et al. (2022) suggests that camels in Egypt are infected with novel *Babesia* species, where they are able to detect *Babesia* sp. *Mymensingh* in one camel in Egypt (Salman et al. 2022). In Egyptian camels, only a few documented about the occurrence of *Babesia* spp. of zoonotic importance. *Babesia microti* is one of the most important species of babesiosis infecting humans (Homer et al. 2000). *Babesia microti* (Apicomplexa: Piroplasmida) is an emerging cause of tick-borne disease with significant public health implications in Asia, Europe, and North America. The main reservoirs of *B. microti* are rodents and their associated ticks, which are more adaptable and resistant to habitat change (Goethert 2021; Hussain et al. 2021; Westblade et al. 2017). The risk of human babesiosis produced by *B. microti* is connected with the presence and prevalence of its tick vectors (Rodgers and Mather 2007). Recent researchers have studied the prevalence of *B. microti* in different tick spp. worldwide. *B. microti* have been found in *Ixodes scapularis*, *I. ricinus*, *I. persulcatus*, and *I. ovatus* (Tuvshintulga et al. 2015). *B. microti* has been recognized as a genetically diverse complex group, generally clarified into the US, Munich, Kobe, and Hobetsu (or Ostu) types (Karnchanabanthoeng et al. 2018; Wei et al. 2020). Only one study in Egypt (Halayeb and Shalateen) detected *B*. *microti* in camels (El-Alfy et al. 2022; Rizk 2021). Consequently, it is necessary to research the prevalence of *B. microti* in Egyptian camels to identify geographic regions where humans are at the highest risk of exposure. So, the aim of this study was to identify *Babesia* species, specifically *Babesia microti*, and their genetic diversity in dromedary camels in Egypt and associated hard tick.

## Methods

### Ethics approval and consent to participate

The study was approved according to the guidelines of the Ethical Committee of the Faculty of Veterinary Medicine, Cairo University (Institutional Animal Care and Use Committee), Vet CU. IACUC (Vet CU28/04/2021/303).

### Study area and collection of samples

The study area included 2 Egyptian slaughterhouses in Cairo and Giza. The sample collection was conducted in 2021. A total of 133 one-humped camels of different ages (3–5 years) and sexes (male and females) that appeared healthy were screened for tick infestation. Different hard tick species (*n* = 1596) were collected and placed in labeled tubes individualized per camel.

About 5 mL of whole blood was taken from the slaughtered camels (*n* = 133). All blood samples were put into labeled EDTA-coated tubes and delivered to the laboratory on ice packs within 4 h for DNA extraction.

### Identification of tick

Adult hard ticks were gathered from camels using tweezers into labeled tubes from predilection sites for ticks (perineum, abdomen, thigh, ear, neck, and dewlap) and transported to the laboratory in a dry ice box. Ticks were first given a two-step cleaning with distilled water, rinsed once with 70% ethanol, and then patted dry with tissue. The tick morphologies were identified to the genus level under a stereomicroscope using taxonomic keys (Estrada-Peña et al. 2004) at the Veterinary Medicine Faculty, Cairo University. The size of mouthparts, the color of the body, leg color, presence or absence of the eye, the shape of the scutum, body, coxae one, festoon, and ventral plates were examined.

### Extraction of DNA from tick and blood samples

A single tick that was representative of each species per individual camel was crushed into small pieces in a mortar using liquid nitrogen for DNA extraction. Genomic DNA was isolated according to the manufacturer’s instructions from 200 µL of blood and tick samples with a Thermo Scientific GeneJET Genomic DNA Purification Kit (Thermo Fisher, Darmstadt, Germany). Genomic DNA was stored at − 20 °C until use.

### Molecular detection of tick species and Babesia species

Molecular identification of tick species was performed using the *COX1* gene and amplified according to the technique described by Abdullah et al. (2016).

To identify *Babesia* spp. from blood samples and ticks, a conventional PCR was used to amplify the *18S rRNA* gene (Casati et al. 2006). The amplification reaction was 12.5 µL of cosmo Taq DNA Polymerase Master Mix (Willowfort, UK) in a total volume of 25 μL, 3 μL of the extracted DNA as a template, and 1 µL of 10 pmol of each primer. The details of primer and PCR conditions are shown in Table 1. Positive controls included the genomic DNA of well-known blood parasites, whereas negative controls included nuclease-free water and both were subjected to the same methods.

**Table 1:** Primer sequences and PCR conditions

Target gene	Primer sequences (5′-3)	Thermal cycles	(bp)	References
*cox*1	CO1-F: GGAACAATATATTTAATTTTTGG CO1-R: ATCTATCCCTACTGTAAATATATG	94 °C, 5 min; 30 cycles (94 °C 1 min; 45 °C, 1 min; 72 °C, 1 min), 72 °C, 10 min	732–820 bp	(Abdullah et al. 2016)
18SrRNA	BJ1: GTC TTG TAA TTG GAA TGATGG BN2: TAG TTT ATG GTT AGG ACT ACG	94 °C, 10 min; 35 cycles (94 °C 1 min; 55 °C, 1 min; 72 °C, 2 min), 72 °C, 5 min	411–452 bp	(Casati et al. 2006)
β-Tubulin	BmTubu93F: GAYAGYCCCTTRCAACTAGAAAGAGC BmTubu897R: CGRTCGAACATTTGTTGHGTCARTTC	95 °C, 10 min; 35 cycles (95 °C, 30 s; 58 °C, 1 min; 72 °C, 1 min 30 s), 72 °C, 10 min	551 bp	(Li et al. 2020)
	BmTubu192F: ACHATGGATTCTGTTAGATCYGGC BmTubu782R: GGGAADGGDATRAGATTCACAGC	94 °C, 10 min; 45 cycles (94 °C 30 s; 61 °C, 30 s; 72 °C, 1 min), 72 °C, 10 min		

*Babesia microti* were identified by nested PCR. Two specific primer sets were used to detect ß-tubulin genes of *B. microti* (shown in Table 1): BmTubu93F and BmTubu897R for the first round and BmTubu192F and BmTubu783R for the second round (Li et al. 2020, Zamoto-Niikura et al. 2012). The reaction was performed in a total volume of 25 μL consisting of 3 µL of template DNA from each tick and blood genomic DNA, 12.5 µL of cosmo Taq DNA Polymerase Master Mix (Willowfort, UK), and 1 µL of 10 pmol of each primer forward and reverse. The PCR products were electrophoresed on 1.5% agarose gel, and a DNA marker and a BERUS 100 bp DNA ladder (Willowfort, UK) were run simultaneously. Positive results for *Babesia* were indicated by the detection of bands with the same size of obtained positive controls.

### Sequencing and phylogenetic analysis

Random selected PCR positive samples were sequenced unidirectionally using primers BN2 and BmTubu192F to confirm the presence of *Babesia* spp. and *Babesia microti*, respectively. The PCR products were purified using a QIAquick PCR Product Purification Kit (Qiagen, Hombrechtikon, Switzerland). Purified PCR products were sequenced using the BigDye Terminator V3.1 sequencing kit (Applied Biosystems, Waltham, MA), and the nucleotide sequences obtained were deposited in GenBank. The acquired nucleotide sequences were compared to those in the public domains using the NCBI-BLAST server and then imported into the BioEdit version 7.0.1.4 program for multiple alignments using the BioEdit Clustal W tool. The MEGA software version X conducted the maximum likelihood method’s phylogenetic analysis based on ß-tubulin genes.

### Statistical analysis

Data was analyzed with PASW Statistics, version 18.0. software (SPSS Inc., Chicago, IL, USA). A chi-square (*χ*^2^) test was used to test the prevalence of different tick genera. Significance was considered at a *P* value less than 0.05.

## Results

### Identification of ticks

Three genera (*Hyalomma*, *Amblyomma*, and *Rhipicephalus*) and twelve hard tick species were identified from (133) *Camelus dromedarius* by using the *COX1* gene, including *Hyalomma dromedarii*, *H. marginatum*, *Amblyomma hebraeum*, *H. excavatum*, *Hyalomma anatolicum*, and *Rhipicephalus annulatus*, and there were few *Amblyomma testudinarium*, *Amblyomma lepidum*, *Amblyomma variegatum*, *Rhipicephalus pulchellus*, *Amblyomma cohaerens*, and finally *Amblyomma gemma*. The prevalence of tick genera collected from camels is shown in Table 2.

**Table 2:** The prevalence of tick genera collected from camels

Tick genera	Total genera	*P* value
*Hyalomma*	1358 (85.1%)^a^	< 0.0001
*Amblyomma*	207 (13.0%)^b^	
*Rhipicephalus*	31 (1.9%)^c^	
Total no. collected	1596	

^a^^,^^b,c^Different superscripts indicate significance (*X*^2^, *P* < 0.05)

The accession numbers of *COX1* gene sequences of identified ticks were deposited in GenBank as OK484562, OQ154976, OL818342, OK340836, OL908962, OQ154978, OQ154975, OQ154974, OQ154972, and OQ154973.

### Molecular detection of Babesia species

In this study, camel blood samples and ticks were screened for the presence of *Babesia* spp. using the 18S rRNA gene. *Babesia* spp*.* were detected in 2.3% (3/133) of blood samples from apparently healthy camels. None of the tick samples tested positive for *Babesia* spp. by 18S rRNA gene (Table 3).

**Table 3:** Molecular detection of *Babesia* spp. and *Babesia microti* in ticks and blood DNA

Type of samples	Total examined	*Babesia* spp.	*B. microti*
		No. (%)	No. (%)
Blood samples	133	3 (2.3)	9 (6.8)
Tick samples	1596	0 (0)	3 (0.2)

### Nested PCR for the detection of Babesia microti in blood and tick samples

After nested PCR targeting the β-tubulin gene, 6.8% (9/133) blood samples and three ticks were positive for *B. microti* (Table 3). The positive ticks included 2 *Rhipicephalus annulatus* and 1 *Amblyomma cohaerens.*

### Sequencing and phylogenetic analyses

The amplified PCR products were subject to DNA sequencing. Six PCR products were sequenced, including 2 for positive blood samples targeting the 18 s rRNA gene, 2 for positive blood samples targeting the ß-tubulin gene, and 1 each for *Amblyomma cohaerens* and *Rhipicephalus annulatus*. The gene sequences were submitted to GenBank and the accession numbers are OP363092, OL912841, OM144475, OM144476, OM103425, and OM103427.

**Fig. 1:**
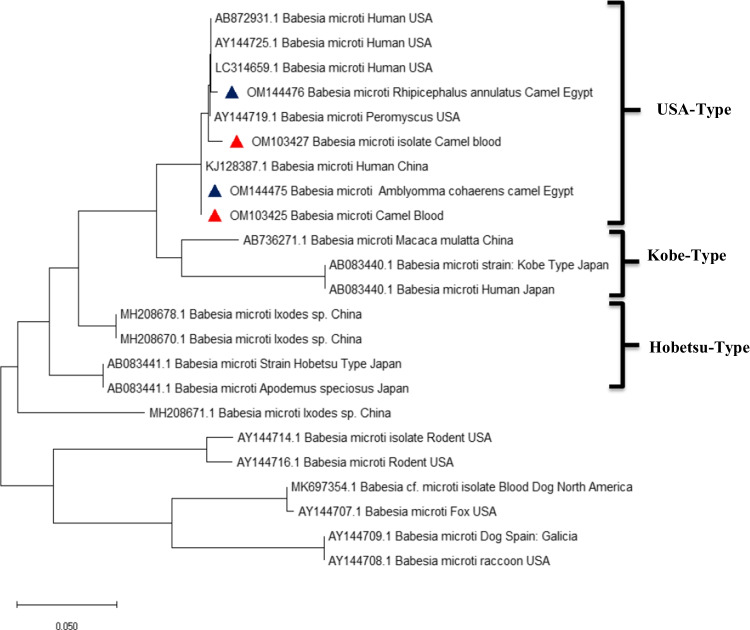
Phylogenetic tree based on the beta-tubulin gene sequences of *B. microti*. The trees were constructed and analyzed by the maximum likelihood method. A red and blue small triangle indicate the new sequences provided by the present study

All strains of *B. microti* isolated from camel’s blood and ticks (*Amblyomma cohaerens* and *Rhipicephalus annulatus*), based on the β-tubulin gene, were closely related to the USA and China strains in human blood (AB872931.1, AY144725.1, and LC314659.1) (Fig. 1).

## Discussion

Tick infestations have a significant impact on the health and productivity of camels, resulting in a high economic loses. The highest prevalent tick genera were the *Hyalomma* genus (85.1%), followed by the *Amblyomma* genus (13.0%) and finally the *Rhipicephalus* genus (1.9%).

The current study detected *Babesia* infection in 2.3% of the camel blood samples based on the 18S rRNA gene. These findings are different to previous studies that recorded an overall prevalence of *Babesia* (*B. bovis* and *B. bigemina*) being 18.43% in Matrouh Governorate, Egypt (El-Naga and Barghash 2016), and 74.5% in Sudan (Ibrahim et al. 2017). Saudi Arabia showed a 13.2% infection rate in camels, while in Iran, it was 6.56% (Khamesipour et al. 2015, Swelum et al. 2014). Nonetheless, the camels are considered an accidental host infected with Babesia and may act as a maintain host for ticks which can transmit the parasite to another host.

One limitation of our research, the expected specificity of the two sets of PCR primers used (18S rRNA primers) was low because these primers are too general and will pick up most types of *Babesia* and *Theileria*. We suggested a more specific set of primers to use for the detection of ß-tubulin genes of *B. microti* by nested PCR.

The *B. microti* ß-tubulin gene was detected in 6.8% of camel’s blood by nested PCR, and this result is lower than the estimated prevalence of *B. microti* in the blood of camels being 11.97% (17/142) in Halayeb and Shalateen in Egypt (Rizk 2021). Only one previous study in Egypt was able to detect *B. microti* in camels; these results highlight the neglected *B. microti* transmission cycle in Egypt.

Fragment of the ß-tubulin gene of *B. microti* was amplified in *Rhipicephalus annulatus* (*n* = 2) and *Amblyomma cohaerens* (*n* = 1) by nested PCR in this investigation. *Babesia microti* was found in *Ixodes ricinus* from small ruminants in Turkey and pets in Belgium (Aydin et al. 2015; Lempereur et al. 2011). It has also been found in China in *Ixodes persulcatus*, *Haematopus longicornis*, and *Haemaphysalis concinna* (Fang et al. 2015, Wei et al. 2020). Whereas ticks of the genus *Ixodes* are considered the primary vector of *B. microti* (Yabsley and Shock 2013), more research must be conducted on other tick species to determine the vector competence of *B. microti* in Egypt.

In the current study, phylogenetic analysis of the ß-tubulin gene of *B. microti* showed a genetically closer relationship of our isolated sequence with similarly related species in the USA and China that were isolated from human blood. This evidenced that US-type *B. microti* were prevalent in Egyptian camels. *B. microti* sensu stricto (or US-type) parasites are probably the most significant parasites due to their impact on global public health as the primary cause of human babesiosis (Goethert 2021). Our study brings light to the presence of *B microti* in Egypt that pose a threat to human health. Therefore, physicians consider it when making a diagnosis.

## Conclusion

*B. microti* US type was detected in different hard ticks (*Rhipicephalus annulatus* and *Amblyomma cohaerens*) and the blood of camels. Further research must be done to clarify the possible role of camels and their parasitic tick species in the enzootic cycle for babesiosis transmission in Egypt*.*

## Data Availability

All the data generated or analyzed in this study are included in this published article.

## References

[CR1] Abdullah HH (2016). Morphological and molecular identification of the brown dog tick *Rhipicephalus sanguineus* and the camel tick *Hyalomma dromedarii* (Acari: Ixodidae) vectors of Rickettsioses in Egypt. Veterinary World.

[CR2] Aydin MF, Aktas M, Dumanli N (2015). Molecular identification of *Theileria* and *Babesia* in ticks collected from sheep and goats in the Black Sea region of Turkey. Parasitology Research.

[CR3] Casati, S. et al., 2006. Presence of potentially pathogenic *Babesia* sp. for human in *Ixodes ricinus* in Switzerland, Annals of Agricultural and Environmental Medicine, 13, 16841874

[CR4] CDC (2012). Babesiosis surveillance-18 states, 2011, MMWR. Morbidity and Mortality Weekly Report.

[CR5] El-Alfy E-S (2022). Molecular Epidemiology and Species Diversity of Tick-Borne Pathogens of Animals in Egypt: A Systematic Review and Meta-Analysis. Pathogens.

[CR6] El-Naga TRA, Barghash S (2016). Blood parasites in camels (*Camelus dromedarius*) in Northern West Coast of Egypt. J. Bacteriol. Parasitol.

[CR7] Estrada-Peña A (2004). Ticks of domestic animals in the Mediterranean region.

[CR8] Fang L-Q (2015). Emerging tick-borne infections in mainland China: an increasing public health threat. The Lancet Infectious Diseases.

[CR9] Goethert HK (2021). What *Babesia microti* is now. Pathogens.

[CR10] Gratz, N., 2006. Tick-borne disease of the USA and Canada, Vector-and Rodent-Borne Diseases in Europe and North America, 185–188

[CR11] Herwaldt BL (2011). Transfusion-associated babesiosis in the United States: a description of cases. Annals of Internal Medicine.

[CR12] Homer MJ (2000). Babesiosis. Clinical Microbiology Reviews.

[CR13] Hussain S (2021). A review of zoonotic babesiosis as an emerging public health threat in Asia. Pathogens.

[CR14] Ibrahim, A.M., Kadle, A.A., and Nyingilili, H.S., 2017. Microscopic and molecular detection of camel piroplasmosis in Gadarif State, Sudan, Veterinary Medicine International, 2017, 10.1155/2017/9345231 PMC5331311 28293445

[CR15] Kalani H, Fakhar M, Pagheh A (2012). An overview on present situation Babesiosis and Theileriosis and their distribution of ticks in Iran. Iranian Journal of Medical Microbiology.

[CR16] Karnchanabanthoeng A (2018). Babesia occurrence in rodents in relation to landscapes of mainland Southeast Asia. Vector-Borne and Zoonotic Diseases.

[CR17] Khamesipour F (2015). Determination of the presence of *Babesia* species in blood samples of cattle, camel and sheep in Iran by PCR. Archives of Biological Sciences.

[CR18] Kim TY (2021). Molecular evidence of zoonotic *Babesia* species, other than *B. microti*, in *Ixodid* ticks collected from small mammals in the Republic of Korea. Veterinary Medicine and Science.

[CR19] Lempereur L (2011). First molecular evidence of potentially zoonotic *Babesia microti* and *Babesia* sp. EU1 in Ixodes ricinus ticks in Belgium. Vector-Borne and Zoonotic Diseases.

[CR20] Li Y (2020). Molecular detection of tick-borne pathogens harbored by ticks collected from livestock in the Xinjiang Uygur Autonomous Region, China. Ticks and Tick-Borne Diseases.

[CR21] Mirahmadi H (2022). Prevalence of camel babesiosis in southeast of Iran. Veterinary Medicine and Science.

[CR22] Motevalli Haghi SM (2014). An overview on different diagnostic methods for Babesiosis. Journal of Mazandaran University of Medical Sciences.

[CR23] Randolph SE, Rogers DJ (2010). The arrival, establishment and spread of exotic diseases: patterns and predictions. Nature Reviews Microbiology.

[CR24] Rizk MA (2021). Molecular detection of *Babesia microti* in one-humped camel (*Camelus dromedarius*) in Halayeb and Shalateen, Halayeb, Egypt. Egyptian Veterinary Medical Society of Parasitology Journal (EVMSPJ).

[CR25] Rodgers SE, Mather TN (2007). Human *Babesia microti* incidence and *Ixodes scapularis* distribution, Rhode Island, 1998–2004. Emerging Infectious Diseases.

[CR26] Salman, D. et al., 2022. Molecular survey of Babesia, Theileria, Trypanosoma, and Anaplasma infections in camels (Camelus dromedaries) in Egypt, Parasitology International, 90, 102618 10.1016/j.parint.2022.102618 35777654

[CR27] Swelum AA (2014). Clinical and laboratory findings associated with naturally occurring Babesiosis in dromedary camels. Bulletin of the Veterinary Institute in Pulawy.

[CR28] Tuvshintulga B (2015). The PCR detection and phylogenetic characterization of *Babesia microti* in questing ticks in Mongolia. Parasitology International.

[CR29] Vannier E, Krause PJ (2012). Human babesiosis. New England Journal of Medicine.

[CR30] Wei C-Y (2020). High prevalence of *Babesia microti* in small mammals in Beijing. Infectious Diseases of Poverty.

[CR31] Westblade LF (2017). *Babesia microti*: from mice to ticks to an increasing number of highly susceptible humans. Journal of Clinical Microbiology.

[CR32] Yabsley MJ, Shock BC (2013). Natural history of zoonotic *Babesia*: role of wildlife reservoirs. International Journal for Parasitology: Parasites and Wildlife.

[CR33] Zamoto-Niikura A (2016). *Ixodes persulcatus* ticks as vectors for the *Babesia microti* US lineage in Japan. Applied and Environmental Microbiology.

[CR34] Zamoto-Niikura A (2012). Detection of two zoonotic *Babesia*
*microti* lineages, the Hobetsu and US lineages, in two sympatric tick species, Ixodes ovatus and *Ixodes*
*persulcatus*, respectively, in Japan. Applied and Environmental Microbiology.

